# Influence of Human Jaw Periosteal Cells Seeded β-Tricalcium Phosphate Scaffolds on Blood Coagulation

**DOI:** 10.3390/ijms22189942

**Published:** 2021-09-14

**Authors:** Marbod Weber, Felix Umrath, Heidrun Steinle, Lukas-Frank Schmitt, Lin Tzu Yu, Christian Schlensak, Hans-Peter Wendel, Siegmar Reinert, Dorothea Alexander, Meltem Avci-Adali

**Affiliations:** 1 Department of Thoracic and Cardiovascular Surgery, University Hospital Tuebingen, Calwerstraße 7/1, 72076 Tuebingen, Germany; marbod.weber@uni-tuebingen.de (M.W.); heidrun.steinle@uni-tuebingen.de (H.S.); christian.schlensak@med.uni-tuebingen.de (C.S.); hans-peter.wendel@med.uni-tuebingen.de (H.-P.W.); 2 Department of Oral and Maxillofacial Surgery, University Hospital Tübingen, Osianderstr. 2–8, 72076 Tübingen, Germany; Felix.Umrath@med.uni-tuebingen.de (F.U.); Lukas-frank.schmitt@student.uni-tuebingen.de (L.-F.S.); lgmolly24@gmail.com (L.T.Y.); siegmar.reinert@med.uni-tuebingen.de (S.R.); dorothea.alexander@med.uni-tuebingen.de (D.A.)

**Keywords:** jaw periosteal cells, bone tissue engineering, biomaterial, β-tricalcium phosphate, hemocompatibility

## Abstract

Tissue engineering offers auspicious opportunities in oral and maxillofacial surgery to heal bone defects. For this purpose, the combination of cells with stability-providing scaffolds is required. Jaw periosteal cells (JPCs) are well suited for regenerative therapies, as they are easily accessible and show strong osteogenic potential. In this study, we analyzed the influence of uncoated and polylactic-co-glycolic acid (PLGA)-coated β-tricalcium phosphate (β-TCP) scaffolds on JPC colonization and subsequent osteogenic differentiation. Furthermore, interaction with the human blood was investigated. This study demonstrated that PLGA-coated and uncoated β-TCP scaffolds can be colonized with JPCs and further differentiated into osteogenic cells. On day 15, after cell seeding, JPCs with and without osteogenic differentiation were incubated with fresh human whole blood under dynamic conditions. The activation of coagulation, complement system, inflammation, and blood cells were analyzed using ELISA and scanning electron microscopy (SEM). JPC-seeded scaffolds showed a dense cell layer and osteogenic differentiation capacity on both PLGA-coated and uncoated β-TCP scaffolds. SEM analyses showed no relevant blood cell attachment and ELISA results revealed no significant increase in most of the analyzed cell activation markers (β-thromboglobulin, Sc5B-9, polymorphonuclear (PMN)-elastase). However, a notable increase in thrombin-antithrombin III (TAT) complex levels, as well as fibrin fiber accumulation on JPC-seeded β-TCP scaffolds, was detected compared to the scaffolds without JPCs. Thus, this study demonstrated that besides the scaffold material the cells colonizing the scaffolds can also influence hemostasis, which can influence the regeneration of bone tissue.

## 1. Introduction

The repair of small and large bone defects is of key interest in oral and maxillofacial surgery. Thus, molecular, as well as cell-based therapies are applied for the engineering of new bone tissue. In most studies, mesenchymal stromal cells (MSCs) derived from bone marrow have been used for regenerative bone formation [1,2]. These cells have a multipotential differentiation capacity into osteoblasts, adipocytes, and chondrocytes [3,4]. MSCs derived from the periosteal tissue are well accessible during routine maxillofacial surgeries [5]. Jaw periosteal cells (JPCs) show strong multipotency [6] and higher proliferation and self-renewal rates compared to bone marrow-derived MSCs [7,8]. In addition, JPCs exhibit reliable osteogenic differentiation while showing a lower chondrogenic and adipogenic differentiation capacity compared to bone marrow-derived MSCs [8]. Consequently, JPCs represent an optimal alternative for bone tissue engineering [9,10,11,12]. 

Besides cellular components, biomaterials used as scaffolds for cells play a decisive role in bone repair [13]. The scaffolds should be biocompatible, support cell attachment, provide stability, and have osteoconductive and osteoinductive properties. For this purpose, different scaffold materials were used, such as hydroxyapatite [14], low-temperature calcium phosphate cement [15], bioactive glass [16], polylactide acid [17], and β-TCP [18]. Calcium phosphate bioceramics have been reported to possess osteoconductive and osteoinductive characteristics due to their structural and chemical similarity to bone tissue, and they can facilitate the osteogenic differentiation of mesenchymal stem cells [19]. β-TCP also offers good structural stability during biodegradation and new bone formation [20]. Moreover, during degradation, large quantities of calcium (Ca^2+^) and phosphate ions (PO_4_^2−^) are released, which are essential inorganic salts for new bone formation [21]. Due to these advantages, β-TCP scaffolds are frequently used in the field of oral and maxillofacial surgery [22,23]. Furthermore, these materials for bone regeneration can be functionalized with cell-binding ligands to improve cell adhesion on their surface [24,25]. It has been demonstrated that a thin coating with poly-lactic-co-glycolic acid (PLGA) can improve the mechanical stability and biocompatibility of β-TCP scaffolds and increase the adhesion and proliferation of JPCs [26].

Immediately after implantation into the bone defects, scaffolds/implants come into close contact with blood, which can lead to the adsorption of plasma proteins on their surface. Plasma proteins can trigger coagulation via the intrinsic pathway, activation of leukocytes, adhesion and activation of platelets [27]. This blood–biomaterial interaction leads to the provisional matrix formation and influences subsequent host reactions to the implants. In the case that scaffolds are colonized with cells, resident cells also come into close contact with blood after the implantation. As a complex biological system, interactions between blood components and JPCs cultivated three-dimensionally on the scaffolds cannot be excluded and also need to be examined in detail. Thus, in this study, we evaluated the interaction of fresh human blood with PLGA-coated and uncoated β-TCP scaffolds seeded with undifferentiated or osteogenically induced JPCs. Hemocompatibility tests were performed according to ISO 10993-4 guidelines. 

## 2. Results

### 2.1. Detection of JPCs on Uncoated and PLGA-Coated β-TCP Scaffolds 

**Figure 1:**
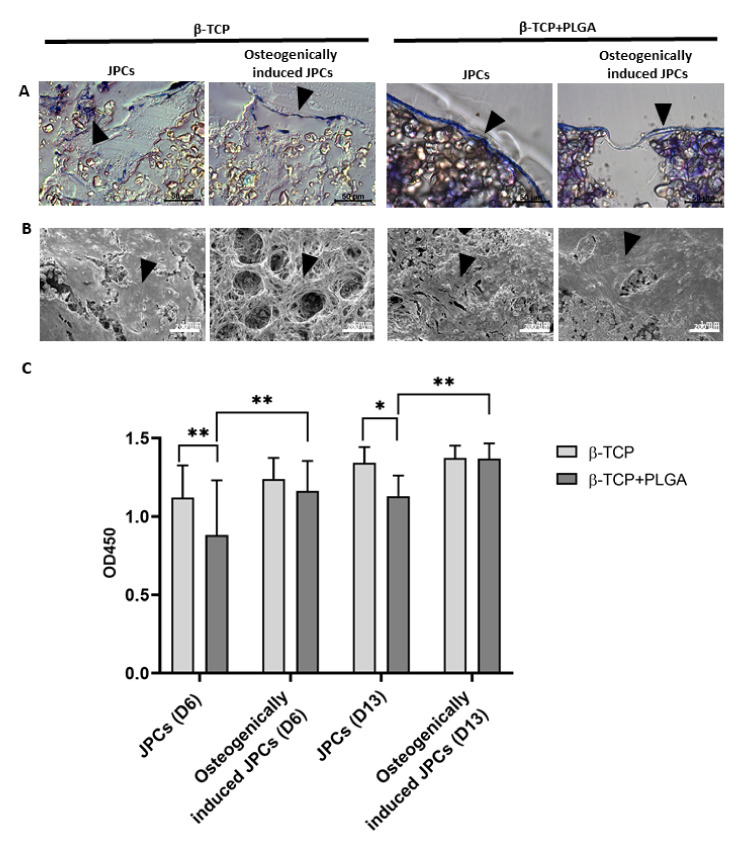
Detection of JPCs on uncoated or PLGA-coated β-TCP scaffolds with and without osteogenic induction and analysis of cell viability. 5 × 10^4^ JPCs were seeded and cultivated for 15 days with or without osteogenic induction medium. (**A**) Histological sections of β-TCP scaffolds with or without PLGA coating were stained with toluidine blue. Arrows indicate toluidine blue-stained cells in histological sections. (**B**) Overview and magnified SEM images of JPCs and osteogenically induced JPCs on β-TCP scaffolds with or without PLGA coating. Black arrows indicate cells on the scaffolds. (**C**) Cell viability was determined by measuring metabolic activity using the colorimetric EZ4U assay. The conversion of tetrazolium salts to formazan derivatives by living cells was measured photometrically at 450 nm. Results are shown as the mean + SD (*n* = 3). Statistical differences were determined using two-way ANOVA (* *p* < 0.05, ** *p* < 0.01).

Uncoated β-TCP scaffolds showed a rough porous surface, while the PLGA-coated scaffolds showed a smooth and less porous structure (Supplementary Figure S1). The β-TCP scaffolds with and without PLGA coating were seeded with 5 × 10^4^ JPCs and cultured either in hPL10 maintenance medium or osteogenic induction medium and after 15 days, histological sections were generated. Using toluidine blue staining, cells were detected in sections of PLGA-coated as well as uncoated β-TCP scaffolds (Figure 1A) with more intense staining in PLGA-coated β-TCP scaffolds. The presence of cells in the scaffolds was also confirmed by SEM (Figure 1B). Depending on the surface structure of the scaffolds, a confluent layer of cells was detected on smooth scaffold surfaces, but also porous surfaces with cavities contained incorporated cells. JPCs cultivated on PLGA-coated β-TCP scaffolds without osteogenic induction at days 6 and 13 showed a slightly lower cell viability compared to the JPCs on uncoated scaffolds (Figure 1C). However, no significant differences in cell viability were detected with osteogenically induced JPCs at days 6 and 13. The osteogenic induction of JPCs seeded on PLGA-coated β-TCP scaffolds led to improved cell viability in contrast to JPCs without osteogenic induction at days 6 and 13. 

### 2.2. Osteogenic Differentiation of JPCs and Cell Mineralization on β-TCP Scaffolds

**Figure 2:**
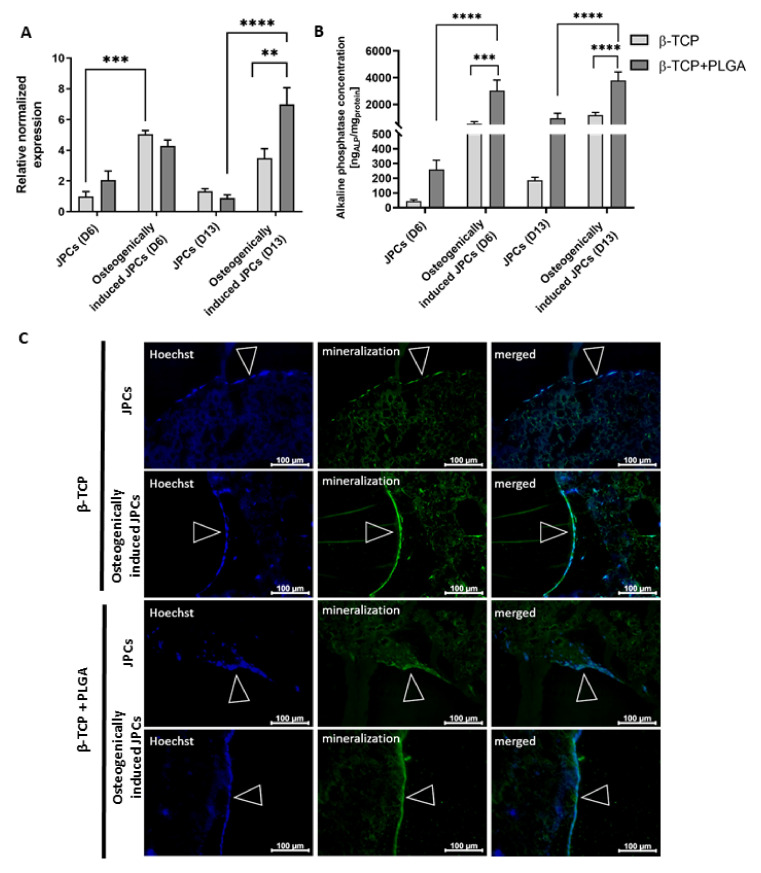
Osteogenic differentiation of JPCs seeded on uncoated and PLGA-coated β-TCP scaffolds. (**A**) Detection of ALP gene expression in JPCs cultivated for 6 and 13 days on uncoated or PLGA-coated β-TCP scaffolds with or without osteogenic induction using qRT-PCR. The mRNA levels were normalized to GAPDH and the results are presented relative to JPCs without osteogenic induction on day 6. Results are shown as the mean + SEM (*n* = 3). (**B**) Detection of alkaline phosphatase activity of JPCs cultivated on uncoated or PLGA-coated scaffolds with or without osteogenic induction medium using the Sensolyte pNPP Alkaline Phosphatase Colorimetric Assay kit. ALP concentrations were normalized to total protein concentrations, which were determined using a BCA assay. Results are shown as the mean + SEM (*n* = 3). Statistical differences were determined using two-way ANOVA (** *p* < 0.01, *** *p* < 0.001, **** *p* < 0.0001). (**C**) Detection of mineralization on day 15 in uncoated or PLGA-coated β-TCP scaffolds with and without osteogenic induction using OsteoImaging mineralization assay. Nuclei were stained blue using Hoechst dye. The green fluorescent staining showed cell mineralization. Arrows indicate confluent cell layers on β-TCP scaffolds.

JPCs were seeded onto β-TCP scaffolds with and without PLGA coating and cultivated with or without osteogenic induction medium. After 6 and 13 days of cultivation, osteogenic differentiation of JPCs was assessed by investigating ALP expression using real-time qRT-PCR, ALP activity, and hydroxyapatite formation (Figure 2). 

After 6 days of osteogenic induction, significantly increased expression of ALP transcript levels was detected in JPCs cultivated on uncoated β-TCP scaffolds compared to JPCs without osteogenic induction (Figure 2A). After 13 days of cultivation on PLGA-coated β-TCP scaffolds, significantly higher ALP transcript levels were detected in JPCs with osteogenic induction compared to the JPCs without osteogenic induction. The osteogenically induced JPCs on PLGA-coated scaffolds also showed significantly higher ALP transcript levels compared to the JPCs on uncoated β-TCP scaffolds. The osteogenic stimulation of JPCs on PLGA-coated β-TCP scaffolds resulted in significantly increased ALP activity (Figure 2B). Furthermore, a significantly increased ALP activity was detected at day 6 and 13 in osteogenically stimulated JPCs seeded on PLGA-coated scaffolds compared to those seeded on uncoated scaffolds. 

Furthermore, the hydroxyapatite produced by the cells was detected after 15 days of cultivation using the OsteoImage mineralization assay (Figure 2C). An enhanced and continuous mineralization layer was detected in scaffolds cultivated in osteogenic induction medium compared to the scaffolds cultivated in the standard hPL10 maintenance medium, which showed only sporadic mineralization. Thereby, the successful osteogenic differentiation of JPCs was demonstrated in PLGA coated and uncoated β-TCP scaffolds. 

### 2.3. Interaction of β-TCP Scaffolds with Human Blood 

β-TCP scaffolds with or without cells were incubated for 90 min at 37 °C with human whole blood to analyze the impact of β-TCP scaffolds and seeded cells on different stages of the hemostatic system. 

#### Analysis of Blood Cell Counts

**Figure 3:**
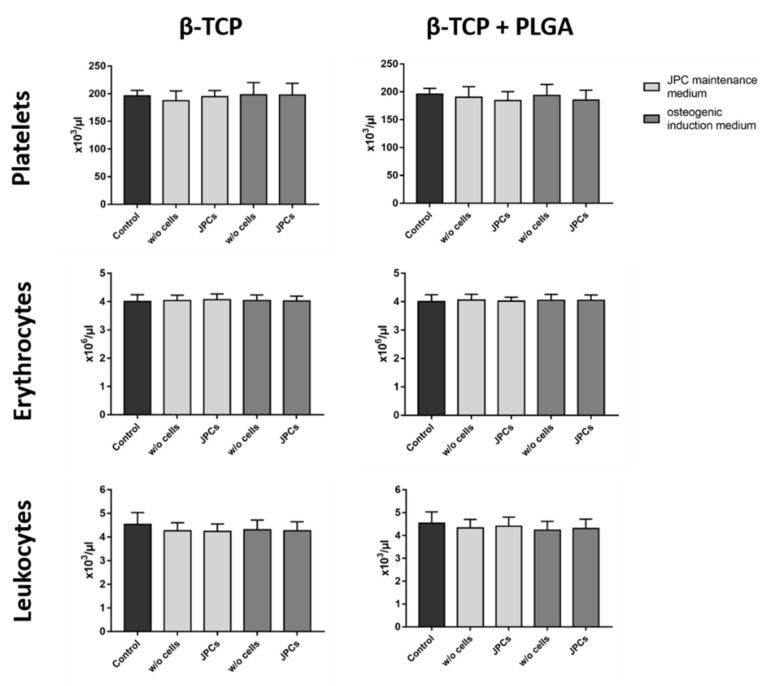
Analysis of blood cell counts after the incubation of blood with β-TCP scaffolds seeded with and without JPCs. PLGA-coated or uncoated β-TCP scaffolds with or without JPCs and with or without osteogenic induction were incubated for 90 min at 37 °C with fresh human blood and the numbers of platelets, erythrocytes, and leukocytes were analyzed. Whole human blood without scaffolds served as a control. Results are shown as the mean + SD (*n* = 3). Statistical differences were determined using one-way ANOVA for repeated measurements followed by Bonferroni’s multiple comparison test.

The interaction of whole blood with biomaterial surfaces can lead to the activation of platelets and leukocytes as well as to the adhesion of these cells to the surface of scaffolds. These interactions can lead to decreased cell numbers in the analyzed blood samples. Therefore, the cell counts were measured before and after the incubation of scaffolds with blood. Performed analyses showed no significant differences in the numbers of platelets, erythrocytes, and leukocytes of the control blood without scaffolds compared to the scaffolds with or without cells and with or without osteogenic induction (Figure 3). 

### 2.4. Analysis of Activation Markers

#### 2.4.1. Activation of Leukocytes

**Figure 4:**
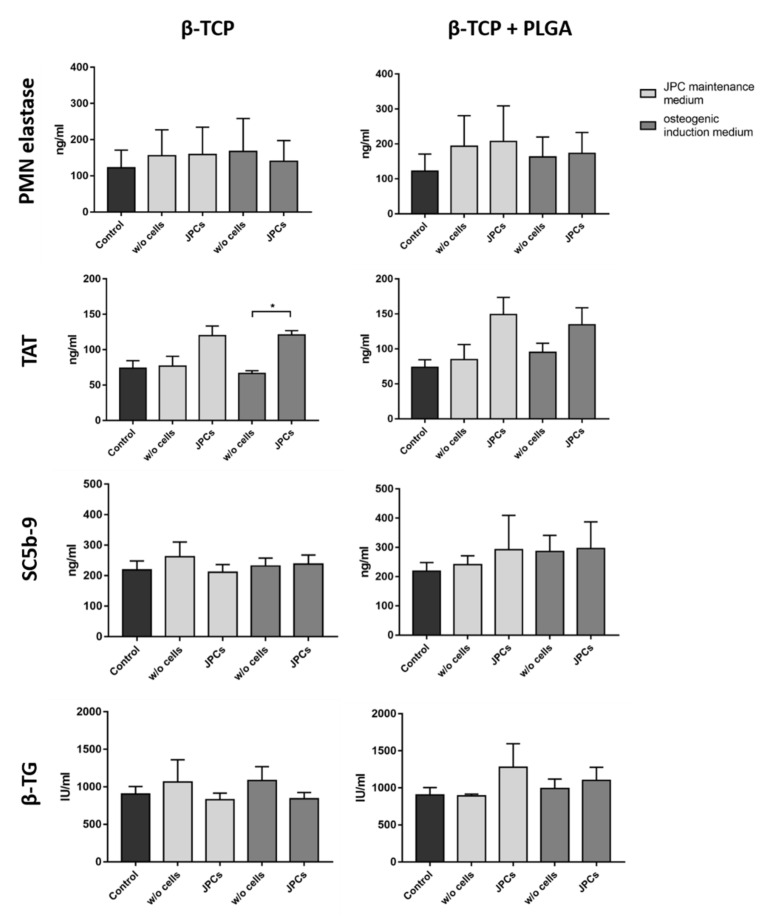
Analysis of hemocompatibility after the incubation of β-TCP scaffolds with and without cells. Analysis of PMN elastase, TAT, SC5b-9, and β-TG concentrations in plasma after the incubation of PLGA-coated or uncoated β-TCP scaffolds with or without JPCs and with or without osteogenic induction for 90 min at 37 °C with fresh human blood. Blood samples without scaffolds served as controls. Results are shown as the mean + SD (*n* = 3). Statistical differences were determined using Kruskal–Wallis tests followed by Dunn’s multiple comparison tests (PMN elastase) or one-way ANOVA followed by Bonferroni’s multiple comparison tests (* *p* < 0.05).

The contact of blood with artificial surfaces can trigger inflammatory processes, which can lead to the activation of leukocytes, such as polymorphonuclear (PMN) leukocytes. This can result in the release of the proteolytic enzyme PMN elastase. In this study, incubation of blood with scaffolds alone or with scaffolds colonized with cells did not significantly alter the PMN elastase concentration compared to the control group (Figure 4). Thus, the tested scaffolds and cells did not activate an inflammatory reaction in the analyzed blood samples.

#### 2.4.2. Activation of the Coagulation System

The interaction of plasma proteins with artificial surfaces can lead to the activation of the intrinsic coagulation pathway. This results in the conversion of prothrombin into thrombin, which in turn leads to the formation of a dense fibrin network. Excessive fibrin formation is counteracted by the coagulation inhibitor antithrombin III, which neutralizes thrombin by forming TAT. Thus, TAT concentration in plasma serves as a marker for the detection of coagulation activation. Scaffolds seeded with JPCs resulted in an increased TAT plasma concentration compared to the scaffolds without cells (Figure 4). In particular, the β-TCP scaffolds seeded with JPCs and cultivated in an osteogenic induction medium led to significantly higher TAT levels. The scaffolds without cells showed similar TAT values as the control. 

#### 2.4.3. Activation of the Complement System

The complement system, which consists of more than 30 proteins, is part of the innate immune system. The contact of foreign surfaces with blood can activate the complement cascade and leads to the generation of the terminal complement complex SC5b9 complex. As a final complex, SC5b-9 is well suited as an indicator of complement activation. The incubation of scaffolds with and without cells in human blood did not induce complement activation (Figure 4). 

#### 2.4.4. Activation of Platelets 

The activation of platelets leads to the release of β-TG, which is stored in alpha granules of platelets. The incubation of uncoated or PLGA coated β-TCP scaffolds with and without cells for 90 min in whole blood did not lead to an increased β-TG concentration (Figure 4). Furthermore, the cultivation of JPCs in osteogenic or maintenance medium did not influence measured β-TG levels in plasma.

### 2.5. SEM Analyses of the β-TCP Scaffolds after Blood Contact

**Figure 5:**
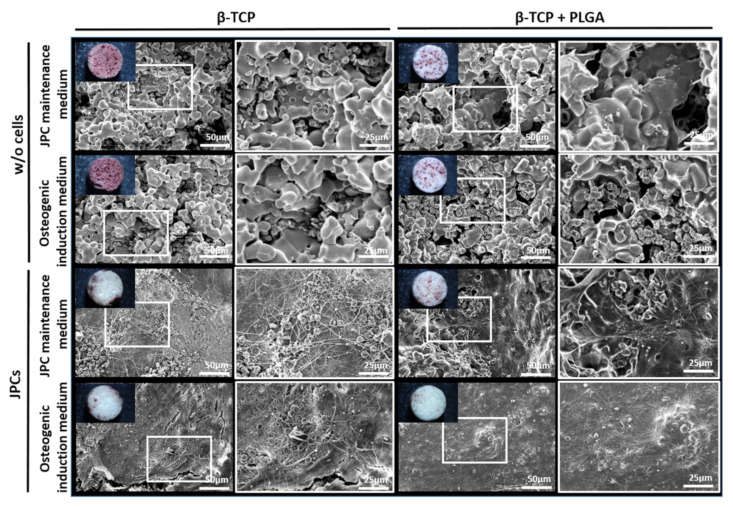
Overview and representative SEM images of β-TCP scaffolds seeded with osteogenically induced and untreated JPCs after the incubation with human blood. SEM images of uncoated and PLGA-coated scaffolds after 90 min of incubation with human whole blood in the rotation model. White frames are showing regions with increased magnification.

SEM was performed to visualize potential fibrin deposits and the attachment of platelets to the scaffolds. The overview images of uncoated β-TCP scaffolds without cells showed an intense red coloration (Figure 5). In contrast, PLGA-coated scaffolds without cells showed reduced red accumulations in the cavities of the scaffold and cell-seeded scaffolds were shown to be largely white with only a few red accumulations. SEM images of the scaffolds without cells also showed accumulations of erythrocytes in the cavities of the scaffold. Thus, the visible red color could be caused by the entrapped erythrocytes in the cavities of the scaffolds. JPC seeded scaffolds showed small accumulations of fibrin networks. Below those fibrin fibers, dense JPC layers were visible, which partly covered the cavities and might be responsible for the reduced entrapment of erythrocytes and red coloration of the scaffolds. A confluent cell layer was detected on the surface of PLGA-coated and uncoated scaffolds cultivated in osteogenic induction medium and reduced fibrin networks. 

## 3. Discussion

The insertion of implants results in tissue injury and direct contact with blood, which can lead to the activation of platelets, leukocytes, and coagulation [27]. A provisional matrix is formed that influences the subsequent host responses to implants. In addition to the scaffold material, the cells seeded on the scaffolds may also have an impact on hemocompatibility. In this study, we used human fresh blood to analyze the impact of PLGA-coated or uncoated β-TCP scaffolds seeded with untreated or osteogenically induced JPCs on hemocompatibility according to ISO 10993-4 for the biological evaluation of medical devices. 

JPCs represent an excellent stem cell type for the repair of small and large bone defects in oral and maxillofacial surgery. The combination of stability-providing scaffolds with cells showing osteogenic potential can be suitable for bone regenerative therapies. Furthermore, the use of PLGA-coated β-TCP scaffolds in combination with JPCs can increase the structural stability of used scaffolds. In addition, the degradation of β-TCP leads to the release of inorganic material such as calcium (Ca^2+^) and phosphate ions (PO_4_^2−^), which have been shown to support mineralization in osteoblast-like cells [21]. In previous studies, PLGA coating resulted in increased JPC proliferation rates [26]. Furthermore, PLGA can be used for biofunctionalization with growth and/or differentiation factors to improve the biofunctionality of the scaffold [28,29].

In this study, coating of β-TCP scaffolds with PLGA resulted in scaffolds with a smoother and less porous structure than uncoated β-TCP scaffolds. However, both scaffold types proved to be suitable substrates for cell adhesion and supported periosteal cell growth. SEM images showed that uncoated and PLGA-coated β-TCP scaffolds were uniformly covered with cells. Successful osteogenic differentiation of JPCs on β-TCP scaffolds was confirmed by significantly increased ALP gene expression levels and activity as well as an increased hydroxyapatite deposition (mineralization) compared with constructs seeded with JPCs cultivated in standard hPL10 medium. The herein performed ALP gene expression and ALP activity analyses indicate that the cultivation of JPCs on PLGA coating led to increased osteogenic cell differentiation. 

The incubation of PLGA-coated or uncoated β-TCP scaffolds seeded with JPCs or osteogenically induced JPCs with whole fresh blood showed no reduction in blood cell counts compared to the control samples without scaffolds. Furthermore, no inflammatory reactions (PMN elastase), activation of the complement system (SC5b-9), or activation of thrombocytes (β-TG) were detected. Although statistically significant only with osteogenically induced JPCs on uncoated β-TCP scaffolds, increased TAT levels, indicative of coagulation activation, were detected when scaffolds were seeded with periosteal cells. The activation of the coagulation cascade can lead to the generation of thrombin and subsequently to the formation of a fibrin network. SEM analyses also confirmed the formation of a fibrin network with entrapped erythrocytes on the surface of the cell-seeded β-TCP scaffolds. In contrast, erythrocytes without the formation of a fibrin network were detected mainly in the cavities of scaffolds without seeded cells. 

The coagulation system can be activated via the contact activation (intrinsic) or the tissue factor (extrinsic) pathway [30]. Previous studies reported that human bone marrow- and periodontal ligament-derived MSCs can lead to thromboembolism after intravenous infusion in mice [31] and intracoronary application in pigs [32]. Intensive investigations revealed that the thromboembolic complications were triggered by the strong expression of the tissue factor on the surface of MSCs and the activation of blood coagulation via the extrinsic pathway [33]. 

During implantation, injury of the vascularized tissue leads to an immediate development of a provisional matrix at the implant site. The generated fibrin network provides a provisional scaffold for wound healing [34]. This matrix also contains plasma fibronectin, which enhances cell adhesion via integrin receptors. Thereby, the migration and adhesion of various cell types, such as fibroblasts and endothelial cells, are stimulated. The formed matrix can also increase the adhesion and activation of platelets. Activated platelets entrapped in the provisional matrix can release various growth factors, such as PDGF, VEGF, bFGF, and TGF-β, which can further enhance the recruitment of fibroblasts, endothelial cells, and immune cells [35]. Attracted endothelial cells improve angiogenesis and fibroblasts produce extracellular matrix proteins, especially collagen. The formation of new blood vessels plays an important role in supplying oxygen and nutrients to the cells of tissue-engineered constructs, and their timely generation is a critical component for the engraftment of constructs and bone healing [36]. Thus, activation of blood coagulation by JPC-populated β-TCP scaffolds compared to JPCs-free scaffolds could further facilitate neovascularization and healing responses resulting in an improved bone healing process.

## 4. Materials and Methods

### 4.1. Coating of β-TCP Scaffolds with PLGA 

The TCP scaffolds were coated with PURASORB PLGA (Corbion Purac, Gorinchem, The Netherlands). Briefly, the polymer (ester terminated, inherent viscosity in CHCl_3_ of 0.4 dL/g) was dissolved in ethyl acetate to obtain a 20% PLGA solution. To perform the PLGA coating, the cylindrical β-TCP scaffolds (Cerasorb M^®^, Curasan, Kleinostheim, Germany) were immersed in the PLGA solution for 15 min and dried in a desiccator. The polymer uptake was measured by weighing and calculating an average of 20 samples.

### 4.2. Cultivation of JPCs

JPCs were isolated from the periosteal tissue of three healthy donors after receiving written informed consent. Periosteal tissue was obtained during interventions at the Department of Oral and Maxillofacial Surgery after approval by the local ethical committee (6182017BO2). 

The cultivation of JPCs was performed in hPL5 medium consisting of DMEM/F12 (Thermo Fisher Scientific Inc., Waltham, MA, USA) supplemented with 5% human platelet lysate (hPL, ZKT Tübingen GmbH, Germany), 100 U/mL penicillin-streptomycin (Lonza, Basel, Switzerland), and 2.5 μg/mL amphotericin B (Biological Industries, Kibbutz Beit Haemek, Israel) in T75 cell culture flasks at 37 °C and 5% CO_2_. The medium was changed every 2–3 days. After reaching 80% confluency, JPCs were detached using TrypleExpress (Thermo Fisher Scientific Inc., Waltham, MA, USA) and the reaction was stopped using hPL5-medium. Afterwards, cells were centrifuged for 5 min at 350× *g* and seeded 1:10 diluted in T75 cell culture flasks.

### 4.3. Seeding of β-TCP Scaffolds with JPCs and Osteogenic Induction

Uncoated or PLGA-coated β-TCP tricalcium phosphate scaffolds were placed in each well of a 96-well plate and preconditioned with 200 μL hPL5-medium at 37 °C. After 1 h, the medium was aspirated and each scaffold was seeded with 5 × 10^4^ JPCs in 50 μL hPL5 medium. The cells were incubated for 2 h at 37 °C to allow cell adherence and then 150 μL medium was added. After 24 h, scaffolds seeded with cells were transferred into a new 96-well plate and 200 μL hPL10 medium (DMEM/F12 containing 10% hPL, 100 U/mL penicillin-streptomycin (Lonza, Basel, Switzerland), 2.5 μg/mL amphotericin B) or osteogenic induction medium (hPL10 containing 0.1 mM L-ascorbic acid 2-phosphate (Sigma-Aldrich, Taufkirchen, Germany), β-glycerophosphate (AppliChem, Darmstadt, Germany), and 4 μM dexamethasone (Sigma-Aldrich)) were added to each scaffold. Cells seeded onto β-TCP scaffolds were cultivated at 37 °C for 15 days with medium changes every 2–3 days. 

### 4.4. Analysis of Cell Viability of JPCs on β-TCP Scaffolds

The EZ4U kit (Biomedica, Vienna, Austria) was used to compare the mitochondrial activity of JPCs cultured on uncoated or PLGA-coated scaffolds. This analysis was performed on day 6 and day 13 of osteogenic differentiation.

The medium was aspirated from the scaffolds and 200 μL of fresh control JPC standard maintenance (hPL5 medium) or osteogenic induction medium and 20 μL of tetrazolium salt substrate was added to each well. After 3 h of incubation at 37 °C and 5% CO_2_, 150 μL were pipetted into a new 96-well plate and absorbance was measured at a wavelength of 450/620 nm using an ELx800 microplate reader (Bio-Tek, Winooski, VT, USA). KC4 software was used for analysis.

### 4.5. RNA Isolation from JPCs Cultured on β-TCP Scaffolds

Cell-seeded scaffolds were transferred to LysingMatrix D Tubes (MP Biomedicals, Santa Ana, CA, USA) containing 600 μL RA1 buffer + TCEP (NucleoSpin RNA Mini Kit, Macherey Nagel, Düren, Germany). Thereafter, samples were homogenized using the FastPrep-24 instrument (MP BiomedicalsSanta Ana, CA, USA). After centrifugation, 500 μL supernatant was transferred into a microcentrifuge tube and total RNA was isolated using the NucleoSpin RNA Mini Kit (Macherey Nagel, Düren, Germany) according to the manufacturer’s instructions.

### 4.6. qRT-PCR Analysis

Total RNA was isolated from JPC seeded β-TCP scaffolds 6 and 13 days after starting the osteogenic induction). As a control, the RNA was isolated from JPC seeded β-TCP scaffolds cultured without osteogenic activators. Complementary DNA (cDNA) was obtained by reverse transcription of 25 ng RNA using the iScript kit (Bio-Rad, Munich, Germany). For the specific amplification of osteogenic markers, the following primers were ordered from Eurofins (Luxemburg, Luxemburg): Alkaline phosphatase (ALP) (forward primer: 5′TGTTCCTGGGAGATGGGTCAG-3′ and reverse primer: 5′CTTGGAGAGGGCCACGAAG-3′). Primers were designed by using the Primer-Blast tool from NCBI [37]. Melting temperatures and self-complementarities were checked using the oligonucleotide properties calculator from Northwestern University Medical School [38]. Real-time qRT-PCR was performed using 300 nM primers, IQ™ SYBR^®^Green Supermix (Bio-Rad), and CFX Connect Real-Time PCR Detection System (Bio-Rad). The amplification of cDNA was performed under the following conditions: 3 min at 95 °C for one cycle, followed by 40 cycles of 95 °C for 15 s, 60 °C for 30 s, and 72 °C for 10 s. Melting curve analysis was performed to ensure the specificity of the PCR products. The samples were run in triplicate with a total volume of 15 μL per well. Levels of mRNA for each gene were normalized to the constitutively expressed internal standard gene glyceraldehyde-3-phosphate dehydrogenase (GAPDH). The results were shown relative to the mRNA levels of JPCs cultivated in a standard maintenance medium (hPL10).

### 4.7. Analysis of Alkaline Phosphatase (ALP) Activity of JPCs in β-TCP Scaffolds

The ALP activity of JPCs on uncoated and coated β-TCP scaffolds was analyzed on day 6 and day 13 of osteogenic differentiation. Four scaffolds were used for each sample. Four scaffolds incubated with the standard JPC maintenance medium during the same time served as controls. 

Analysis was performed using the Sensolyte pNPP alkaline phosphatase colorimetric assay kit (AnaSpec, Fremont, CA, USA). For extraction of cell lysates, scaffolds were crushed using MagNA Lyser Green Beads tubes (Roche Diagnostics, Rotkreuz, Switzerland) and a FastPrep^®^-24 device (MP Biomedicals, Santa Ana, CA, USA). For this purpose, MagNA Lyser Green Beads tubes (Roche Diagnostics, Germany) were first filled with 600 μL lysis buffer and placed on ice. The scaffolds were washed twice with 200 μL assay buffer and four scaffolds of the same condition were transferred into one tube. Subsequently, the scaffolds were disrupted using the FastPrep^®^-24 device (MP Biomedicals, Santa Ana, CA, USA) and the cells were lysed during this process. After cooling on ice, the tubes were centrifuged (10,000× *g* at 4 °C, 10 min) and 400 μL of the supernatant was pipetted into a fresh 2 mL tube. Then, the sample was centrifuged again (2500× *g*, 4 °C, 10 min) and 350 μL of the clear cell lysate was transferred to a fresh 2 mL tube. Samples were diluted 1:5 and 1:10 with assay buffer and standards with a known ALP concentration were prepared. 50 μL of the diluted cell suspensions and standards were pipetted into a 96-well plate and 50 μL of pNPP substrate solution was added. After 30 min of incubation at room temperature, 50 μL of stop solution was added to terminate the reaction. Subsequently, absorbance was measured at a wavelength of 405 nm using an ELx800 plate reader (Bio-Tek, Winooski, VT, USA). 

To account for possible differences in cell numbers on the scaffolds, the determined ALP concentration was normalized to the total protein concentration. This was determined using the Pierce BCA Protein Assay Kit (Thermo Fisher Scientific Inc., Waltham, MA, USA) according to the manufacturer’s instructions. Using an ELx800 plate reader (Bio-Tek, Winooski, VT, USA), the absorbance at a wavelength of 550 nm was measured. KC4 software was used for analysis. Based on the ALP concentration and the total protein concentration, the ALP fraction in the total protein concentration (ng AP/mg protein) was calculated.

### 4.8. Embedding and Generation of Thin Polished Sections of β-TCP Scaffolds Seeded with JPCs

After 15 days of cultivation, the scaffolds seeded with JPCs were washed twice in phosphate-buffered saline (PBS) without Mg^2+^ und Ca^2+^, fixed in 4% paraformaldehyde (PFA), and washed again twice in PBS. Ascending alcohol series (70%, 80%, 96%, 2 × 100% EtOH, 15 min each) and xylene (2 × 100%, 30 min each) were used for dehydration and degreasing. To facilitate polymethyl methacrylate (PMMA) infiltration, the samples were incubated twice for 1 h in absolute acetone. Subsequently, the scaffolds were transferred into 2 mL microcentrifuge tubes and treated overnight at 4 °C with 300 μL of solution A (500 mL destabilized base solution, 25 g PMMA powder, 3 g hardener 1, Technovit 9100 (Kulzer, Wehrheim, Germany)). The next day, samples were embedded in 2 mL embedding solution (9 parts solution A with 1 part solution B (44 mL base solution, 4 mL hardener, and 2.2 mL regulator), Technovit 9100). The embedding solution was cured overnight at −18 °C in the absence of oxygen, and then for 24 h at 4 °C. Subsequently, the samples were transferred for 1 h into a 37 °C water bath for complete polymerization of the embedding solution. 

Embedded scaffolds were cut to sections of 5 μm using an electronic rotary microtome (pfm Rotary 3006 EM; pfm medical AG, Cologne, Germany) with a hard-cutting blade (SH35W Feather microtome blade HP TC; Feather Safety Razor Co., LTD., Osaka, Japan). To stabilize the samples, sections were collected on transparent adhesive tape (tesafilm, Beiersdorf AG, Norderstedt, Germany) and transferred to microscope slides.

### 4.9. Toluidine Blue Staining

First, the sections were acidified with 10% acetic acid for 5 min, washed with distilled water, and dried. In the next step, a drop of the toluidine staining solution (0.1% (*w/v*) toluidine blue O (Sigma-Aldrich) in distilled water) was added and incubated for 15 min. The sections were then rinsed with distilled water and dried overnight and mounted using DePeX (Serva, Heidelberg, Germany) and coverslips.

### 4.10. Analysis of Mineralization 

After 15 days of JPC cultivation and osteogenic stimulation on β-TCP scaffolds, constructs were analyzed via OsteoImage^TM^ mineralization assay kit (Lonza, Basel, Switzerland). Briefly, microtome sections of PMMA (Technovit 9100) embedded scaffolds were first deacrylated and then stained with the OsteoImage Mineralization Assay Kit (Lonza, Walkersville, MD, USA) for the detection of hydroxyapatite. Hoechst 33,342 (PromoKine, Heidelberg, Germany) was used as a nuclear counterstain. 

For deacrylation, microtome sections were placed in a cuvette with 100% xylene for one min and then dried in a drying cabinet at 37 °C for at least 30 min. Next, the staining solution (Staining Reagent diluted 1:100 in Staining Reagent Dilution Buffer, OsteoImage Mineralization Assay)) was added to the samples and incubated for 30 min in the dark. After the washing with OsteoImage Wash Buffer, Hoechst 33,342 nuclear staining solution (1:1000 dilution of stock solution in PBS) was added to the samples for 10 min. After washing and drying, the sections were mounted with glycerol.

### 4.11. Analysis of Hemocompatibility 

#### 4.11.1. Collection of Human Blood

Human whole blood was collected from the antecubital vein of non-medicated healthy volunteers (*n* = 3) via venipuncture in monovettes preloaded with 1 IU/mL sodium heparin (LEO Pharma Inc., Neu-Isenburg, Germany). The blood sampling procedure was approved by the Ethics Committee of the medical faculty at the University of Tuebingen (287/2020BO2) and all subjects gave written informed consent.

#### 4.11.2. Incubation of Scaffolds with Whole Human Blood

β-TCP scaffolds seeded with JPCs were cultivated in vitro for 15 days. The scaffolds were then transferred into 12 mL polypropylene round-bottom tubes (Becton Dickinson, Franklin Lakes, NJ, USA) containing 11 mL blood. Control tubes contained the same amount of fresh blood without scaffolds. The incubation with blood was performed at 37 °C for 90 min using a tube rotator (neoLab, Heidelberg, Germany) with 10 rpm. After 90 min, blood was transferred into monovettes containing ethylenediaminetetraacetic acid (EDTA) (Sarstedt Inc., Nümbrecht, Germany) for the analysis of complement activation and detection of cell numbers. Blood cell numbers were determined using a Micros 60 cell counter (ABX Diagnostics, Montpellier, France). A citrate solution containing monovettes (Sarstedt Inc.) was used to analyze PMN-elastase and TAT. To analyze β-TG concentrations, blood was transferred to citrate-theophylline-adenosine-dipyridamole (CTAD) containing monovettes (BD Biosciences Inc.) and stored for 15 min on ice. EDTA and CTAD monovettes were centrifuged at 2500× *g* for 20 min at 4 °C. Citrate blood monovettes were centrifuged at 1800× *g* for 18 min at RT. The plasma of each sample was shock frozen in liquid nitrogen and stored at −20 °C (EDTA and citrate plasma) or −80 °C (CTAD plasma) until further investigations.

#### 4.11.3. Detection of Activation Markers

Commercially available enzyme-linked immunosorbent assays (ELISA) were used to investigate the hemocompatibility. The PMN-elastase ELISA (Demeditec, Kiel, Germany) was used to detect the activation of leukocytes. The activation of coagulation was detected using TAT ELISA (Enzygnost Micro Assay, Siemens Healthcare, Erlangen, Germany). Furthermore, SC5b-9 ELISA (Osteomedical GmbH, Bünde, Germany) was used to detect the complement activation and platelet activation was determined using β-TG ELISA (ASSERA-CHROM^®^, Diagnostica Stago, Asnieres, France). The ELISAs were performed according to the manufacturer’s instructions.

#### 4.11.4. Scanning Electron Microscopy (SEM)

After the incubation with blood, the β-TCP scaffolds were fixed overnight at 4 °C in 2% glutaraldehyde (Serva, Heidelberg, Germany). Overview images were taken with a Canon reflex camera EOS 450D (Canon, Tokyo, Japan) and a NOVEX RZB-SF stereo microscope (Euromex, Arnhem, The Netherlands). Afterwards, the samples were washed with PBS for 10 min and dehydrated with an ascending ethanol series (40% to 100% ethanol; Merk, Darmstadt, Germany) in 10 min steps. The scaffolds were dried in a critical point drier (Polaron E3100, GaLa Instruments, Bad Schwalbach, Germany). Subsequently, the scaffolds were sputtered with gold-palladium particles (Emitech K550X, GaLa Instruments, Bad Schwalbach, Germany) and analyzed by SEM (Zeiss LEO1430, Zeiss, Oberkochen, Germany). 

### 4.12. Statistical Analysis

Data are shown as the mean + standard deviation (SD) or standard error of the mean (SEM). Means were compared using one or two-way analysis of variance (ANOVA) for repeated measurements followed by Bonferroni’s multiple comparison test or using the Kruskal–Wallis test followed by Dunn’s multiple comparisons test, depending on the distribution of the data. Statistical analyses were performed double-tailed using GraphPad Prism 6.01 (GraphPad Software, La Jolla, CA, USA). Differences of *p* < 0.05 were considered significant.

## 5. Conclusions

In this study, we demonstrated that uncoated and PLGA-coated β-TCP scaffolds are highly suitable for the colonization with JPCs and their further osteogenic differentiation. The osteogenic induction resulted in mineralization and increased expression of and ALP. Cell-free β-TCP scaffolds with and without PLGA coating showed no effect on blood cells, complement, and coagulation activation. In contrast, scaffolds seeded with JPCs led to increased levels of TAT and accumulation of fibrin fibers on the surface of the constructs, presumably through activation of the extrinsic coagulation pathway. These results demonstrated that hemocompatibility can not only be influenced by the scaffold material alone but also by seeded cells inside the scaffolds, which can have an impact on tissue compatibility and the healing process within the bone defect. 

## Data Availability

The analyzed data sets generated during the study are available from the corresponding author on reasonable request.

## Supplementary Materials

The following are available online at www.mdpi.com/article/10.3390/ijms22189942/s1.
