# Supplementary figures and images for: Influence of Human Jaw Periosteal Cells Seeded β-Tricalcium Phosphate Scaffolds on Blood Coagulation
Source: Int J Mol Sci. 2021 Sep 14;22(18):9942. doi: 10.3390/ijms22189942 (PMC8467579; doi:10.3390/ijms22189942)

## Supplementary Data

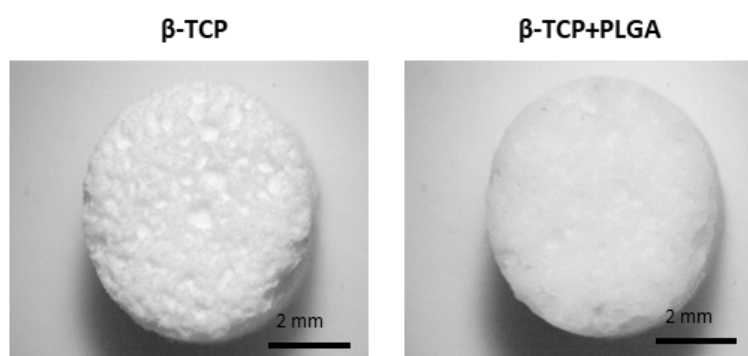

**Figure S1.** Overview images of uncoated and PLGA-coated  $\beta$ -TCP scaffolds.

Supplement: Supplementary file 1 [file ijms-22-09942-s001.zip › ijms-1366033-supplementary.pdf]
